# Recent insights into PERK-dependent signaling from the stressed endoplasmic reticulum

**DOI:** 10.12688/f1000research.12138.1

**Published:** 2017-10-27

**Authors:** Alexander McQuiston, J Alan Diehl

**Affiliations:** 1 Department of Biochemistry and Molecular Biology, Hollings Cancer Center, Medical University of South Carolina, Charleston, SC, USA

**Keywords:** PERK, Unfolded Protein Response, tumorigenesis, protein translation

## Abstract

The unfolded protein response (UPR) is an evolutionarily conserved stress response to intra- and extracellular conditions that disrupt endoplasmic reticulum (ER) protein-folding capacity. The UPR is engaged by a variety of disease conditions, including most cancers as well as both metabolic and neurodegenerative disorders. Three transmembrane transducers—PERK, IRE1, and ATF6—are responsible for activating downstream signaling pathways that mediate the UPR and subsequent stress response pathways. PERK, an ER resident transmembrane protein kinase, initiates both pro-apoptotic and pro-survival signaling pathways. In the context of neoplasia, PERK and its downstream targets alter gene expression that can be both pro- and anti-tumorigenic. In this review, we discuss recent advances in understanding how canonical and non-canonical PERK-mediated signaling pathways influence cell fate, tumor progression, and tumor suppression and avenues for therapeutic intervention.

## Introduction

The endoplasmic reticulum (ER) is the site of post-translational modification, folding, maturation, and secretion for transmembrane and secreted proteins. The rate of protein transport into the ER and its folding capacity fluctuates on the basis of intra- and extracellular conditions and varies among cell type. Cells can adapt to increased nascent protein import into the ER lumen and folding demands by preferentially increasing the overall size of the ER and upregulating translation of chaperone proteins ^1^. However, under stressful conditions, such as tumorigenesis, protein translation exceeds ER folding capacity, resulting in the accumulation of misfolded proteins within the ER. As a result of the accumulation of misfolded proteins, an evolutionarily conserved stress response known as the unfolded protein response (UPR) is activated. The function of the UPR is to either re-establish homeostasis or trigger cell death in order to prevent accumulation of damaged, non-functional cells.

Mammalian cells have three ER resident transmembrane proteins—protein kinase RNA-like ER kinase (PERK), inositol-requiring enzyme 1 alpha/beta (IRE1α/β), and activating transcription factor 6 (ATF6)—that function as signal transducers of the UPR. All three transmembrane proteins contain a single transmembrane domain, luminal domain, and are present at a basal level in their inactive state. PERK and IRE1α also consist of a cytosolic tail that has kinase activity and kinase and ribonuclease activity, respectively. The inactive states of PERK, IRE1α, and ATF6 are characterized by the binding of binding immunoglobulin protein (BiP)/glucose-regulated protein 78 kDa (GRP78) ATPase domain to their luminal domain ^2^. Decreased expression of BiP/GRP78 via repression of its coding gene, *HSPA5*, activates all three UPR sensors ^3^. Furthermore, accumulation of misfolded proteins sequesters BiP/GRP78 away from PERK, IREα, and ATF6. BiP sequestration may occur through the interaction of unfolded proteins, such as CH1. CH1 interacts with the substrate-binding domain of BiP/GRP78 where it potentially triggers the dissociation of BiP/GRP78 from the luminal domains of PERK, IRE1α, and ATF6 ^2, 4^. Ultimately, reduced BiP/GRP78 interaction is essential for activation of PERK, IRE1α, and ATF6.

Sequestration of BiP/GRP78 from the luminal domain of PERK triggers oligomerization and autophosphorylation ^5^. PERK is found in both a dimer and a transient tetramer state, and the tetramer state is a higher state of activation than the dimer. PERK luminal domains oligomerize to form stable dimers and then a helix swap, or the intertwining of two dimers via helical subunit, produces the tetramer configuration ^6^. The tetramer interface is primarily composed of hydrophobic residues that are thought to help stabilize the tetramer structure and increase phosphorylation efficiency ^6^. Transient configuration changes between the dimer and the tetramer may represent an intrinsic form of regulation based on the level of misfolded proteins in the ER lumen. Activated PERK phosphorylates eukaryotic initiation factor 2α (eIF2α), thereby reducing translation initiation for a majority of cellular proteins. However, a select set of mRNAs that typically encode short open reading frames within the 5′ untranslated region (UTR) (upstream open reading frames, or uORFs), such as basic leucine zipper protein family member activating transcription factor 4 (ATF4), are translationally induced ^7^.

Similar to PERK, titration of BiP/GRP78 leads to IRE1α oligomerization and activation via trans-autophosphorylation. In addition to functioning as a protein kinase, the IRE1α cytosolic domain harbors ribonuclease activity which mediates the degradation of ER-localized mRNAs through a process known as regulated IRE1-dependent decay (RIDD). While RIDD triggers decay of both non-coding and coding RNA, IRE1 ribonuclease activity also specifically splices an intron from XBPI mRNA, thereby increasing XBP1 translation. Genes downstream of XBP1s influence protein secretion, cell survival and apoptosis, and DNA damage and repair ^8^. IREα associates with the Sec61 translocon to locate XBP1 unspliced mRNA ^9^. Interestingly, repression of translocon subunits specifically activated IRE1α and leads to XBP1 mRNA splicing equal to that of decreased BiP/GRP78 interaction ^10^. Activation of IRE1α through loss of translocon interaction suggests UPR signaling pathway activation specificity. PDIA6 influence on IRE1 and ATF6 activation independent of BiP, but not PERK activation, further suggests UPR signaling pathway activation specificity ^11, 12^.

ATF6 is a transmembrane transcription factor. ER stress causes export of ATF6 to the Golgi followed by two sequential cleavage events by protease site-1 protease (S1P) and protease site-2 protease (S2P). The two cleavage events expose ATF6’s transcriptionally active cytosolic domain, which translocates to the nucleus. ATF6 also induces XBP1 mRNA, highlighting the cross-talk between UPR pathways ^13^.

The pathways described above represent the canonical ER stress and UPR signaling pathway. In recent years, great strides have been made to better understand the canonical and non-canonical signaling pathways that influence other stress response mechanisms and cell fate and ultimately revealed potential roles of both canonical and non-canonical pathways in disease. In the following sections, we will highlight recent novel findings pertaining to the role of UPR in regulating cell fate following exposure of cells to tumor-associated stresses and the potential for therapeutic development.

## UPR signaling and cell fate

### Signaling through eIF2α

The integrative stress response (ISR) is a complex signaling pathway that is activated by both intracellular and extracellular stressors. Stress-specific protein kinases recognize stress induction and mediate ISR downstream signaling pathways. For example, amino acid deprivation, heme deprivation, viral infection, and misfolded proteins are recognized by GCN2, HRI, PKR, and PERK, respectively ^14^. While there is inherent stress recognition specificity, there is also considerable redundancy among these protein kinases that has been elucidated through gene-specific knockout mice ^15^. Interestingly, the ISR downstream signaling pathways of all four previously mentioned kinases converge on the phosphorylation of eIF2α. Of particular interest in the present review is the PERK-eIF2α UPR signaling pathway.

eIF2α coordinates the formation of eIF2α-GTP-Met-tRNA _i_^Met^, a component of the 43S pre-initiation complex necessary for formation of the 80 S ribosome complex. Formation of the 80S ribosome complex requires hydrolysis of eIF2-GTP to eIF2-GDP, which leads to dissociation of the ribosomal complex for translation termination. The eIF2α guanine exchange factor, eIF2β, is responsible for exchanging GDP for GTP to enable eIF2α-GTP-Met-tRNA _i_^Met^ complex reformation and ultimately re-initiate protein translation ^16^. However, phosphorylation of eIF2α by PERK (HRI, PKR, or GCN2) inhibits eIF2β-dependent exchange of GDP for GTP, thereby preventing reformation of the 43 S pre-initiation complex and subsequent ribosome complex. mRNAs containing uORFs will stall 43S pre-initiation complexes to block translation, but at low concentrations the 43 S pre-initiation complex will skip the uORFs, enabling mRNA translation ^17, 18^.

### PERK mediates pro-death and pro-survival signaling

**Figure 1.**
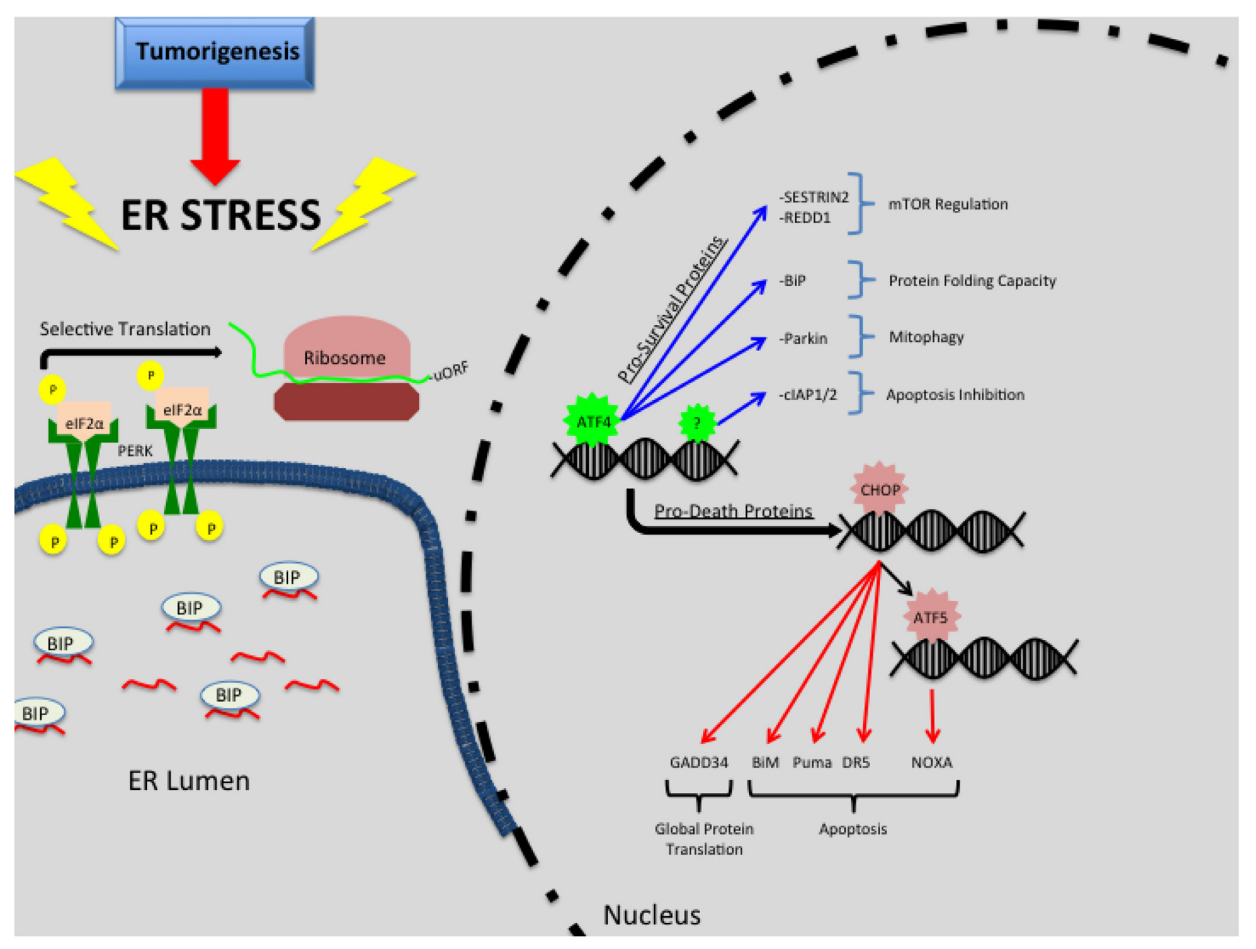
Activation of unfolded protein response and downstream pro-survival and pro-death proteins. Tumorigenesis induces protein synthesis that exceeds the endoplasmic reticulum (ER) folding capacity and increases the number of nascent proteins within the ER lumen leading to ER stress. Sequestration of binding immunoglobulin protein (BiP) from the luminal domain of protein kinase RNA-like ER kinase (PERK) to nascent proteins enables PERK oligomerization, autophosphorylation, and activation. Activated PERK phosphorylates eukaryotic initiation factor 2α (eIF2α) at Ser51 and causes selective translation of proteins containing upstream open reading frames (uORFs). Selectively translated proteins, like transcription factor activating transcription factor 4 (ATF4), directly or indirectly regulate expression of pro-survival and pro-death proteins. Red arrows represent pro-death pathways, and blue arrows represent pro-survival pathways.

PERK-mediated eIF2α phosphorylation directly regulates expression of cell fate–determining genes through several mechanisms. ATF4, which is translationally induced by PERK is the most heavily studied. The ATF4 mRNA contains overlapping uORFs in its 5′ UTR which are necessary or and preferentially ATF4 translation upon phosphorylation of eIF2α ( Figure 1). ATF4 directs transcription of a complex network of genes that ultimately determine cell fate. ATF4 induces expression of multiple ER resident chaperone proteins (such as BiP/GRP78) to increase folding capacity, mediate amino acid metabolism and glutathione synthesis, and increase resistance to oxidative stress ^19^. ATF4 also induces autophagy genes that are important in autophagosome formation and function ^20^. Inhibitors of apoptosis, cIAP1 and 2, are also induced during ER stress in a PERK-dependent but ATF4-independent manner ^21^ ( Figure 1).

A well-known ATF4 pro-death target gene is the transcription factor C/EBP homologous protein (CHOP), which further promotes transcription of pro-death genes ^22^ ( Figure 1). CHOP is directly responsible for inducing expression of two BH3-only pro-apoptotic Bcl-2 family members: Bim and Puma. Bim and Puma mediate cell death by negatively regulating the activity of pro-survival Bcl-2 family members ^23^. Negative regulation of pro-survival Bcl-2 family member activity via Bim and Puma enables Bax/Bak oligomerization mediating the apoptotic release of cytochrome C ^24, 25^. CHOP indirectly induces expression of another BH3-only pro-apoptotic Bcl-2 family member, NOXA, through induction of the transcription factor ATF5 ^26^. Similar to Bim and Puma, NOXA negatively regulates pro-survival Bcl-2 family proteins. Furthermore, CHOP induces expression of death receptor 5 (DR5) which will bind Fas-associated death domain (FADD) independent of the DR5 ligand, Apo2/TRAIL ^27^. FADD transduces pro-death signals by activating caspase 8 ^28^. Interestingly, DR5 mRNA can be degraded via RIDD, highlighting regulatory cross-over between UPR signaling pathways ^27^.

ATF4 also impacts cell fate through modulation of apoptosis inhibitors such as XIAP. Here, transcriptional induction of ubiquitin ligases increases XIAP proteasomal degradation, thereby increasing apoptosis. In this instance, however, induction of ubiquitin ligase and subsequent XIAP proteasomal degradation is CHOP-independent ^29^. ATF4/CHOP induces GADD34, which encodes a protein that directs protein phosphatase 1 (PP1) to eIF2α ^30^. The induction of *Gadd34* creates a negative feedback loop to re-establish global protein translation and can increase protein load during ER stress, exacerbating stress and causing cell death ^31^.

**Figure 2.**
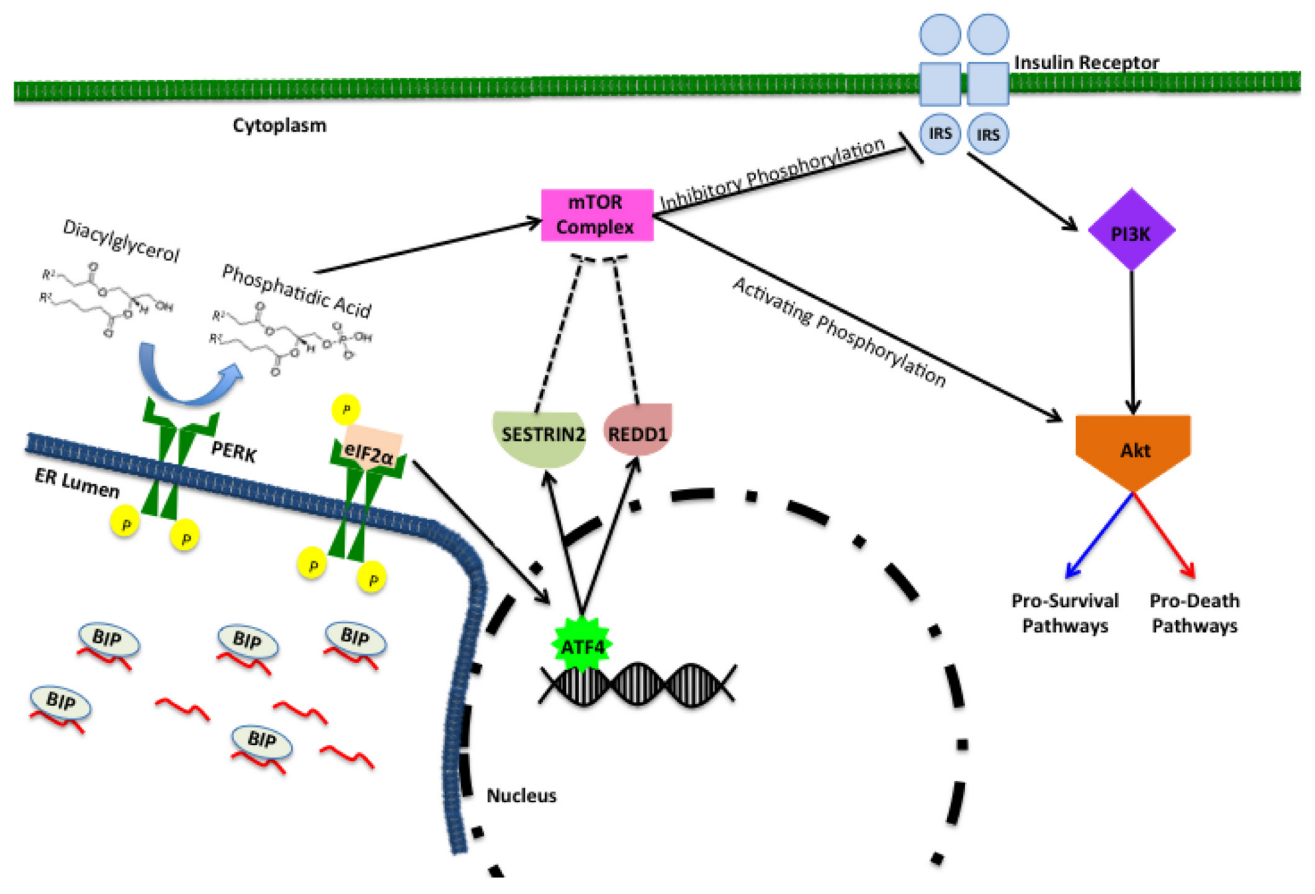
PERK-mediated regulation of the mTOR-PI3K-Akt pathway. Endoplasmic reticulum (ER) stress sequesters ER resident chaperone binding immunoglobulin protein (BiP) away from luminal domain of PERK, leading to PERK activation. Activated PERK contains lipid kinase activity and converts diacylglycerol to phosphatidic acid (PA). PA is instrumental in mammalian target of rapamycin (mTOR) complex formation. mTOR activation can inhibit insulin receptor substrates via phosphorylation and block PI3K-Akt activation. Alternatively, mTOR complex can activate Akt and downstream pathways via phosphorylation. Activating transcription factor 4 (ATF4)-dependent expression of SESTRIN2 and regulated IRE1-dependent decay 1 (RIDD1) can indirectly suppresses mTOR activity (indirect regulation illustrated by dashed lines). Akt-mediated downstream signaling pathways can have both pro-survival and pro-death impacts on cell fate. mTOR, mechanistic target of rapamycin; PERK, protein kinase RNA-like endoplasmic reticulum kinase.

PERK also mediates pro-survival and pro-death signaling through the mechanistic target of rapamycin (mTOR) pathway ( Figure 2). mTOR is a kinase that regulates cell growth and proliferation via nutrient availability and protein translation and is dysregulated in many cancers ^32, 33^. PERK has intrinsic lipid kinase activity that mediates production of the pro-mitogenic phospholipid, phosphatidic acid (PA), via phosophorylation of diacylglycerol ^34^. PA has been implicated in mTOR activation via competition with rapamycin and is essential for mTOR complex (mTORC) formation ^35– 37^. Therefore, PERK’s lipid kinase activity produces PA-mediating mTORC formation and subsequent Akt activating phosphorylation ( Figure 2). Akt downstream signaling pathways are highly complex as they both mediate survival and apoptotic responses ^35, 36^. Another example of PERK-mediated mTOR regulation is the ATF4-dependent expression of DNA damage and development 1 (REDD1) and SESTRIN2, both of which can suppress mTOR ^38– 41^ ( Figure 2). One of mTOR’s functions is to mediate inhibitory phosphorylation of insulin receptor substrate (IRS) docking proteins that activate the PI3K pathway ^42, 43^. Suppression of mTOR prevents inhibitory phosphorylation of IRS docking proteins thereby activating PI3K and subsequent Akt signaling pathways ( Figure 2).

PERK signaling can also play a role in mitochondrial pro-survival signaling ^44^. The ER and mitochondrial membranes are connected via mitochondria-associated ER membranes (MAMs) ^44^. Interestingly, activated PERK has been localized to MAMs, suggesting that PERK’s activation and downstream signaling pathways can influence mitochondrial mediated cell survival ^45^. Furthermore, PERK-ATF4 transcriptionally induces expression of Parkin, a protein that mediates autophagy of mitochondria (mitophagy) ^46, 47^. Mitophagy promotes cell survival by maintaining mitochondrial homeostasis.

### Non-coding RNA mediated pro-death and pro-survival signaling

The role of non-coding RNAs in cell fate has become an area of intense investigation. Two non-coding RNAs—microRNA (miRNA) and long non-coding RNA (lncRNA)—are regulated by the UPR and play significant roles in cell survival and cell death signaling. miRNAs are small regulatory non-coding RNA molecules approximately 22 nucleotides in length with a seven-nucleotide seed sequence that recognizes complementary sequences in target mRNA. Upon recognition, the most miRNAs mediate target mRNA degradation or inhibit their translation. However, some miRNAs can lead to mRNA-specific upregulation ^48^. Advances in high-throughput sequencing technology facilitated the identification of up to 86 differentially expressed miRNAs following ER stress induction ^49^. Much of the miRNA regulation that influences cell fate occurs through the PERK arm of the UPR. Interestingly, one miRNA, miR-204, directly targets and inhibits PERK signaling ^50^. Repression of PERK signaling blocks expression of most genes necessary to overcome ER stress and leads to cell death.

**Figure 3.**
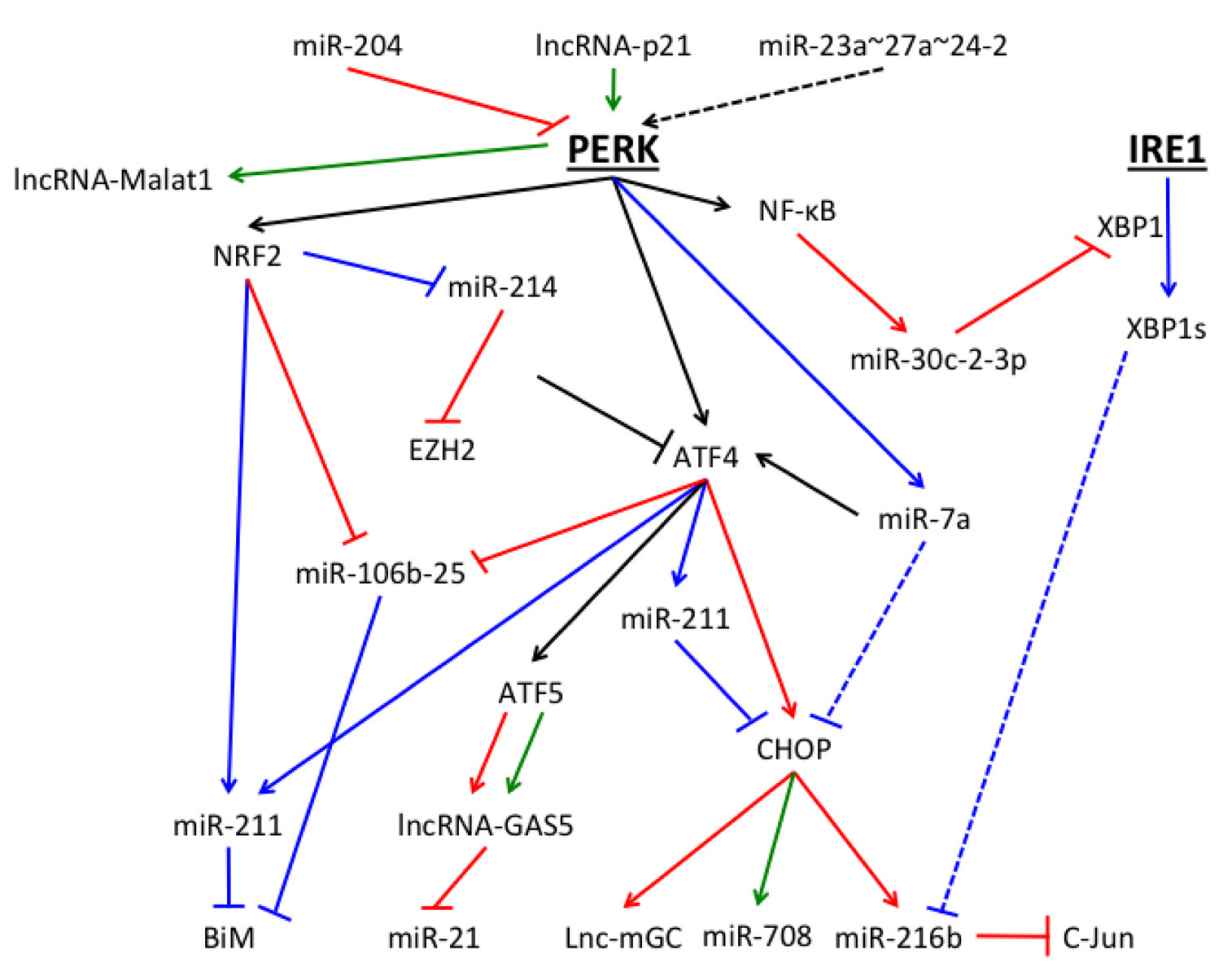
Activation of unfolded protein response and downstream pro-survival, pro-death, and tumorigenic related non-coding RNA. Endoplasmic reticulum (ER) stress induces complex non-coding RNA, both microRNA (miRNA) and long non-coding RNA (lncRNA), regulatory pathways that influence cell fate and tumorigenesis. Two miRNAs—miR-204 and miR023a~27a~24-2—repress and activate protein kinase RNA-like ER kinase (PERK) activity, respectively. Furthermore, multiple miRNAs and lncRNAs are induced or repressed following PERK activation. Black arrows represent pathways that can lead to either pro-survival or pro-death signaling, blue arrows represent pathways that lead to pro-survival signaling, red arrows represent pathways that lead to pro-death signaling, and green arrows represent pathways that influence tumorigenesis. Dotted lines represent indirect regulatory pathways.

Downstream of PERK, ATF4-dependent expression of miR-211 and NRF2-dependent repression of miR-214 promote cell survival ^51, 52^ ( Figure 3). MiR-211 contains a seed sequence targeting the promoter region of *chop* ^51^. Repression of *chop* inhibits pro-apoptotic signaling and promotes cell survival. MiR-214 targets both ATF4 and EZH2. Decreased expression of miR-214 enables ATF4 and EZH2 expression, which increases transcription of pro-survival genes and represses pro-apoptotic protein BiM, respectively ^52^. Cell survival is also promoted by ER stress-induced expression of miR-7a and subsequent indirect repression of pro-apoptotic transcription factor CHOP ^49^. CHOP induces expression of miR-216b, which mediates translational repression of c-Jun and sensitizes cells to ER stress-induced apoptosis ^53^. Interestingly, miR-216b is indirectly regulated by the IRE1 branch of the UPR, suggesting cross-over in miRNA regulation ^53^ ( Figure 3).

miRNAs that are within adjacent regions and transcribed in the same orientation can form miRNA clusters. Most clusters contain two to three miRNAs, and different miRNAs within a cluster can have different targets ^54^. PERK-mediated induction of ATF4 and NRF2 downregulates expression of miR-106b-25 cluster and increases cell death ^55^. Repression of this cluster enhances ER stress-induced apoptosis because miR-106b-25 cluster antagonizes pro-apoptotic protein BiM translation. Furthermore, PERK/NF-κB induces miR-30c-2-3p expression which targets the IRE1-dependent transcription factor XBP1 ^56^. Spliced XBP1 (XBP1s) induces transcription of important pro-survival genes; therefore, decreased XPB1 can induce cell death. Regulation of miR-30c-2-3p also demonstrates cross-talk between arms of the UPR. Interestingly, in contrast to ER stress inducing miRNA expression, miR-23a~27a~24-2 cluster induces ER stress and increases expression of pro-apoptotic proteins CHOP and Bim ^57^ ( Figure 3).

lncRNAs, RNA molecules of more than 200 base pairs, regulate cellular processes at both the transcription and translational level, and some evidence suggests regulation at the post-translational level ^58^. Multiple lncRNAs are regulated under ER stress states and play a role in UPR-mediated cell fate. The lncRNA TUG1 protects hepatocytes from apoptosis by repressing expression of essential UPR proteins BiP/GRP78, PERK, phosphorylated eIF2α, and CHOP ^59^. CHOP induces expression of lnc-mGC, a lncRNA that binds a megacluster of 40 miRNAs and promotes cell death through changes in multiple miRNA-mediated translation regulation pathways ^60^. Furthermore, PERK-dependent ATF4 expression induces expression of ATF5, which subsequently induces expression of GAS5 ^26^ ( Figure 3). GAS5 expression can promote apoptosis by multiple hypothesized mechanisms, including repression of steroid receptor–induced transcriptional activation, inhibition of miR-21, and sensitizing cells to external stressors ^61^. More research is needed to identify UPR-mediated lncRNA and elucidate their highly complex role in determining cell fate.

## ER stress-induced tumor suppression versus tumor progression

Malignant transformation and tumor progression must bypass ER stress, which is induced by factors including aberrant expression of oncogenes and a microenvironment with disordered vasculature that contributes to nutrient restriction, hypoxia, and increased ROS ^62, 63^. Given the propensity of the UPR and PERK to induce pro-survival signaling, significant efforts have been devoted to elucidate its contribution to tumor progression with the underlying assumption that PERK will be pro-tumorigenic; if so, the expectation was tumor addiction to PERK signaling. However, many genetic experiments support both a tumor-suppressive and tumor-promoting function for PERK.

Activation of the potent oncogene, HRAS, in non-cancerous melanocytes increased cellular senescence in a PERK-dependent manner, suggesting that PERK mediates senescence in pre-malignant cells ^64^. Furthermore, some cancer cell lines display decreased PERK-mediated eIF2α signaling, suggesting that PERK activation and CHOP expression may attenuate malignant transformation and progression under certain conditions or in a cell type–dependent manner ^62, 65^. Consistent with this notion, PERK displays characteristics of a haploinsufficient tumor suppressive in melanocytes ^66^. ER stress-induced PERK activation can also influence immunological recognition of malignant cells. For example, PERK activation upregulates the ER chaperone calreticulin, which can be translocated to the cell surface of malignant cells and act as a phagocytic signal for immune cells ^67^.

PERK-dependent non-coding RNAs can also act as tumor suppressors. For example, CHOP-dependent miR-708 targets neuronatin, which decreases intracellular calcium levels, resulting in reduced metastasis ^68, 69^. Likewise, lncRNA-p21 induces ER stress through PERK activation and mediates hepatocellular carcinoma apoptosis ^70^. In addition, CHOP mediates expression of ATF5, which induces expression of lncRNA GAS5, which is pro-apoptotic in different cancers ^61, 71^. Ultimately, PERK activation- or deactivation-mediated tumor suppression signaling may be cancer-specific and further investigation is needed.

PERK-mediated tumor progression can occur at multiple stages in cancer development. PERK activation can help pre-malignant cells cope and survive ER stress conditions enabling neoplastic transformation ^72^. Recent studies have demonstrated the importance of PERK activation in tumor metastasis by mediating pathways that promote tumor cell epithelial-to-mesenchymal transition (EMT) and detachment and invasion ^73– 76^. The PERK-dependent lncRNA Malat1 is a marker in numerous cancers and plays an important role in lung cancer progression and metastasis ^77– 79^. Tumor angiogenesis can be indirectly induced by ATF4-dependent induction of the aryl hydrocarbon receptor and subsequent expression of vascular endothelial growth factor in hepatoblastoma cells ^80^. Interestingly, PERK activation can further promote therapy resistance and resistance to hypoxia ^81^. The dynamic role of PERK-mediated signaling in tumor progression and resistance makes PERK and its downstream pathways attractive for therapeutic intervention.

## Therapeutic potential

The contribution of PERK-dependent gene expression to cell survival stimulated the development of strategies for therapeutic intervention. Recently, a potent PERK inhibitor, GSK2606414, was synthesized and specifically inhibits PERK activation as well as decreases tumor growth in human tumor xenograft mice ^82^. GSK2606414 was later modified to a more pharmacological stable form, GSK2656157, which demonstrated dose-dependent inhibition of human tumor xenograft growth in mice and the potential for further clinical implementation ^83, 84^.

Targeting PERK may not be without consequence. PERK signaling is critical for enabling normal cells to overcome ER stress. PERK also elicits anti-proliferative signals through silencing of G _1_ cyclins and induction of pro-apoptotic pathways ^85, 86^. In addition, while PERK is non-essential in most adult tissues, PERK deletion or inhibition can lead to pancreatic failure and hyperglycemia ^87^. Further investigation of PERK inhibition–associated risks in *in vivo* models is needed to determine therapeutic potential.

Other approaches to therapeutic intervention have demonstrated promising anti-tumor potential. For example, the eIF2α phosphatase complex inhibitor, salubrinal, induced apoptosis, increased chemotherapy efficiency, and restored treatment sensitivity in chemo-resistant cancer cells ^88– 91^. Also, salubrinal treatment in conjunction with a proteasome inhibitor increased apoptosis in leukemic cells ^88^. Inhibition of GRP78 demonstrated anti-angiogenic potential, and indirect inhibition of the PERK/eIF2α/ATF4 pathway inhibited EMT ^92, 93^.

Additionally, two small molecules—guanabenz and Sephin1—have been reported to selectively inhibit the eIF2α phosphatase complex ^94, 95^. Decreased dephosphorylation of eIF2α prolongs global protein translation repression in stressed cells and increases chaperone-folding ability. Guanabenz and Sephin1 blocked eIF2α dephosphorylation by inducing a conformational change that disrupts recruitment of eIF2α to its phosphatase complex ^96^. Efficacy of the aforementioned therapeutics, though promising, may hinder their clinical impact as some have suggested that guanabenz and Sephin1 do not interfere with eIF2α dephosphorylation ^97^. Combinatorial studies of PERK and downstream target inhibitors with other drugs such as proteasome inhibitors may be one approach to increasing efficacy and reducing toxicities.

## Concluding remarks

PERK-mediated signaling pathways are complex and vary between tissue and cell type but play a clear role in cell fate and tumorigenesis. PERK activation and downstream signaling pathways have also been implicated to play a role in other pathologies such as neurodegeneration and diabetes ^87, 98, 99^. The findings presented in this review demonstrate the tremendous progress toward understanding PERK biology. However, while we are beginning to elucidate signals that drive these PERK-mediated responses, there remain significant gaps in our knowledge that will compromise our ability to translate inhibitors successfully into the clinic. Furthermore, the role and mechanisms of ER stress-induced non-coding RNAs remain incompletely understood. Ultimately, a deeper understanding of these remaining questions will help elucidate the role of PERK in different pathologies for more effective therapeutic intervention.
